# Supplementary figures and images for: Protease-associated import systems are widespread in Gram-negative bacteria
Source: PLoS Genet. 2019 Oct 15;15(10):e1008435. doi: 10.1371/journal.pgen.1008435 (PMC6793856; doi:10.1371/journal.pgen.1008435)

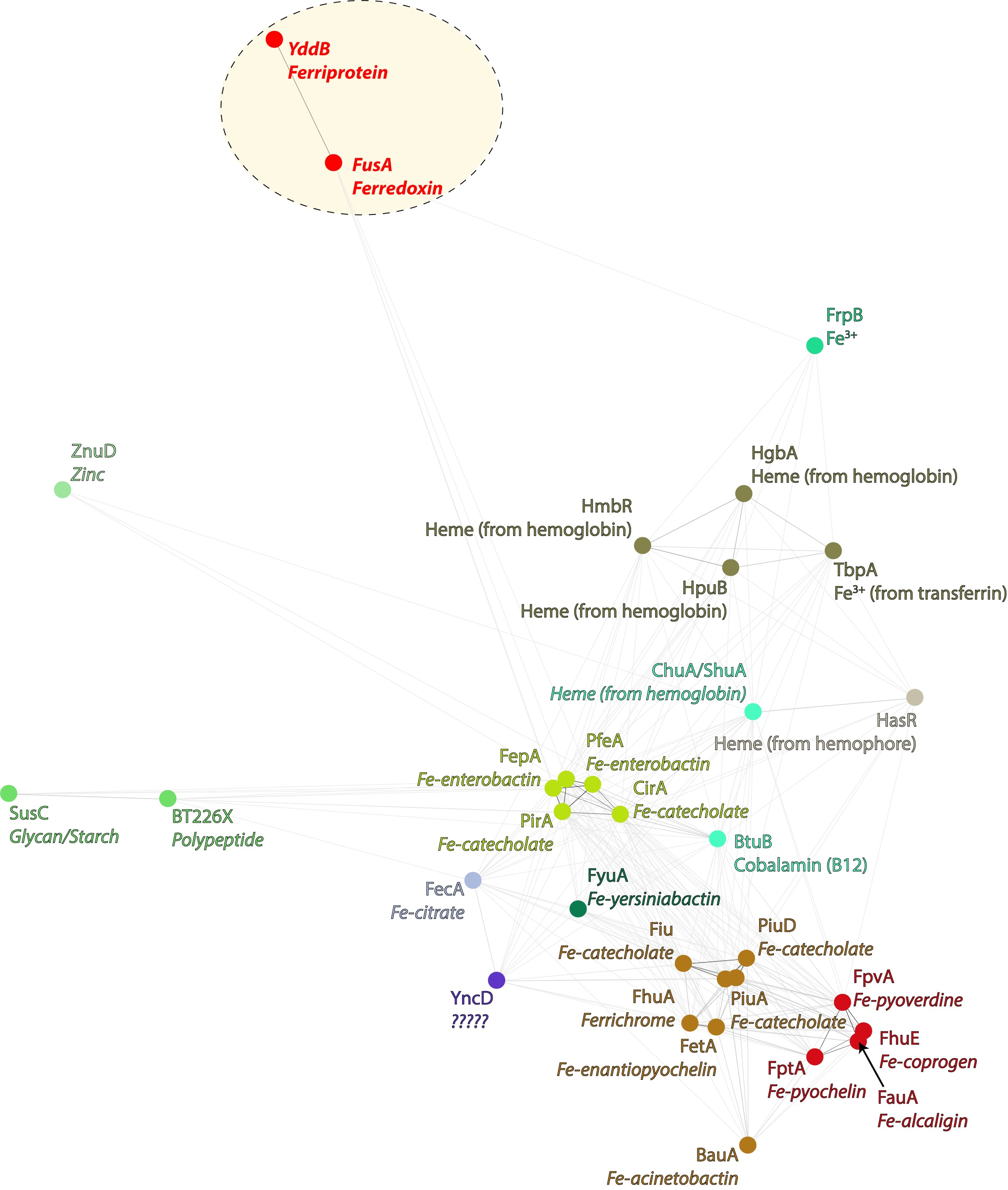

Supplement: S1 Fig — Representative/structurally characterized TonB-dependent transporters were clustered using CLANS, demonstrating that YddB and FusA form a sequence cluster that is similarly distantly related to other TonB-dependent transporters. FusA/YddB are similarly distant to the main group of transporters as the highly divergent SusC family from Bacteroides spp.; further illustrating a distant relationship between FusA and other transporters. Dots represent individual sequences and grey lines represent pairwise similarity relationships. An E-value cut-off of 1e-110 was used for clustering. (TIF) [file pgen.1008435.s001.tif]

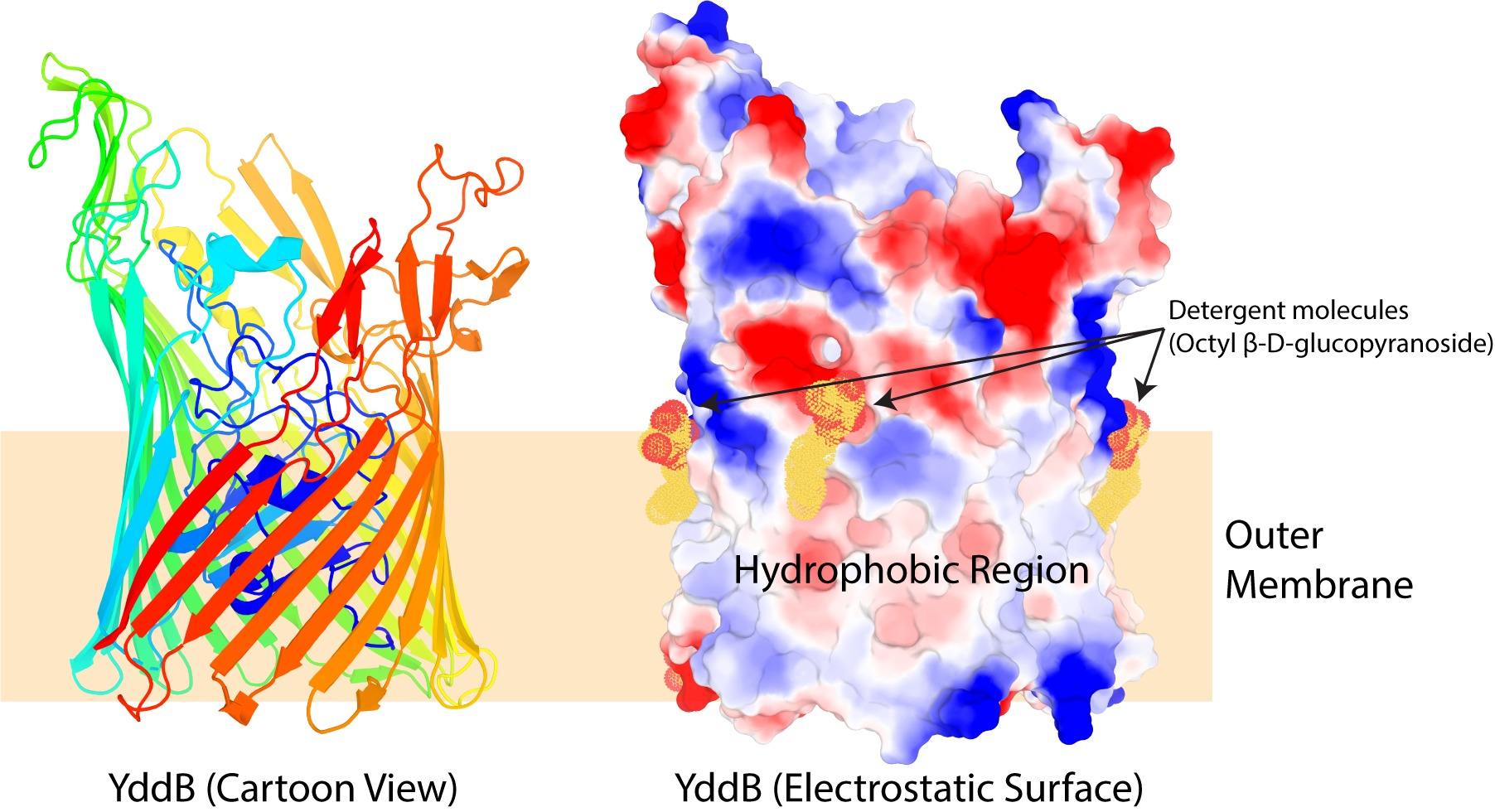

Supplement: S2 Fig — The crystals structure of YddB shown as rainbow cartoon (N-terminus = blue, C-terminus = red) (left), and electrostatic surface (right). The electrostatic surface illustrates the presence of a hydrophobic transmembrane region, which embeds YddB in the membrane. Octyl β-D-glucopyranoisde detergent molecules observed shielding the hydrophobic region in the crystal structure are shown as spheres. (TIF) [file pgen.1008435.s002.tif]

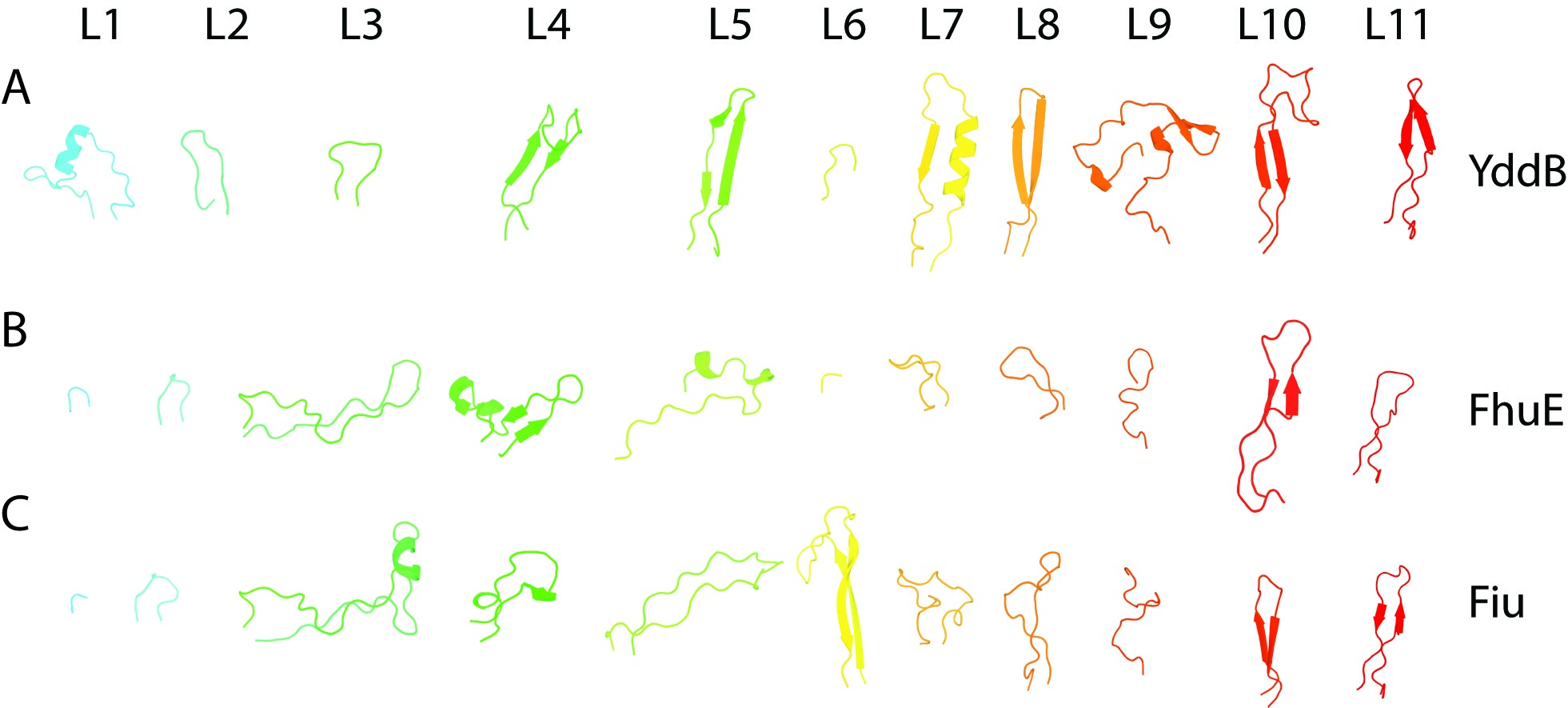

Supplement: S3 Fig — The extracellular loops of YddB (A) are distinct in structure and length from those of FhuE (B) and Fiu (C), transporters for coprogen and catecholate siderophores respectively. (TIF) [file pgen.1008435.s003.tif]

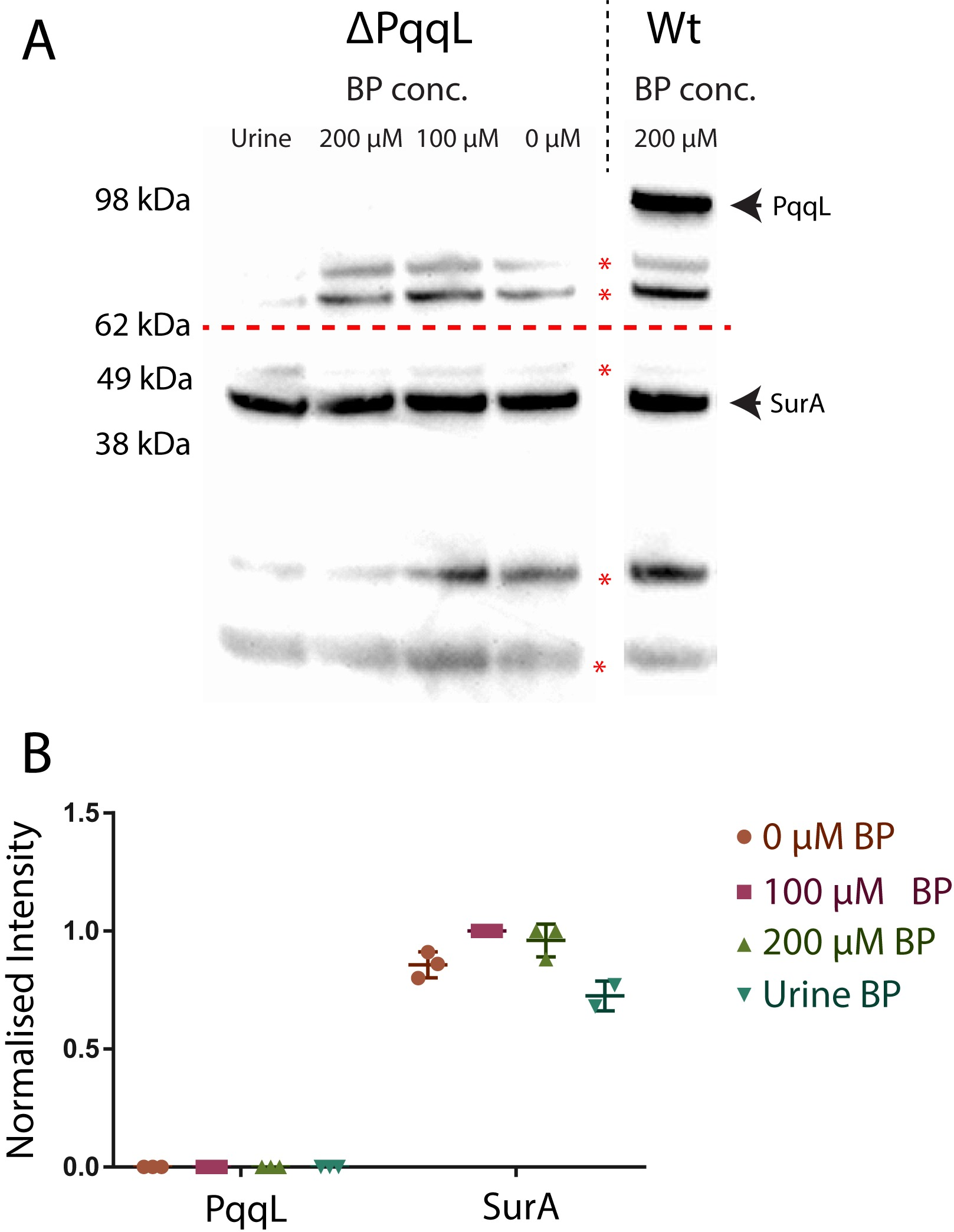

Supplement: S4 Fig — (A) A representative western blot of E. coli BW25113 ΔpqqL whole cells with anti-PqqL (top) and anti-SurA (bottom) in the presence and absence of 2,2’bipyridine, showing no band corresponding to PqqL is detected in this strain. Detection of PqqL in wildtype E. coli BW25113 is shown as a reference. (B) Quantitation of 3 biological replicate of blots of panel A. (TIF) [file pgen.1008435.s004.tif]

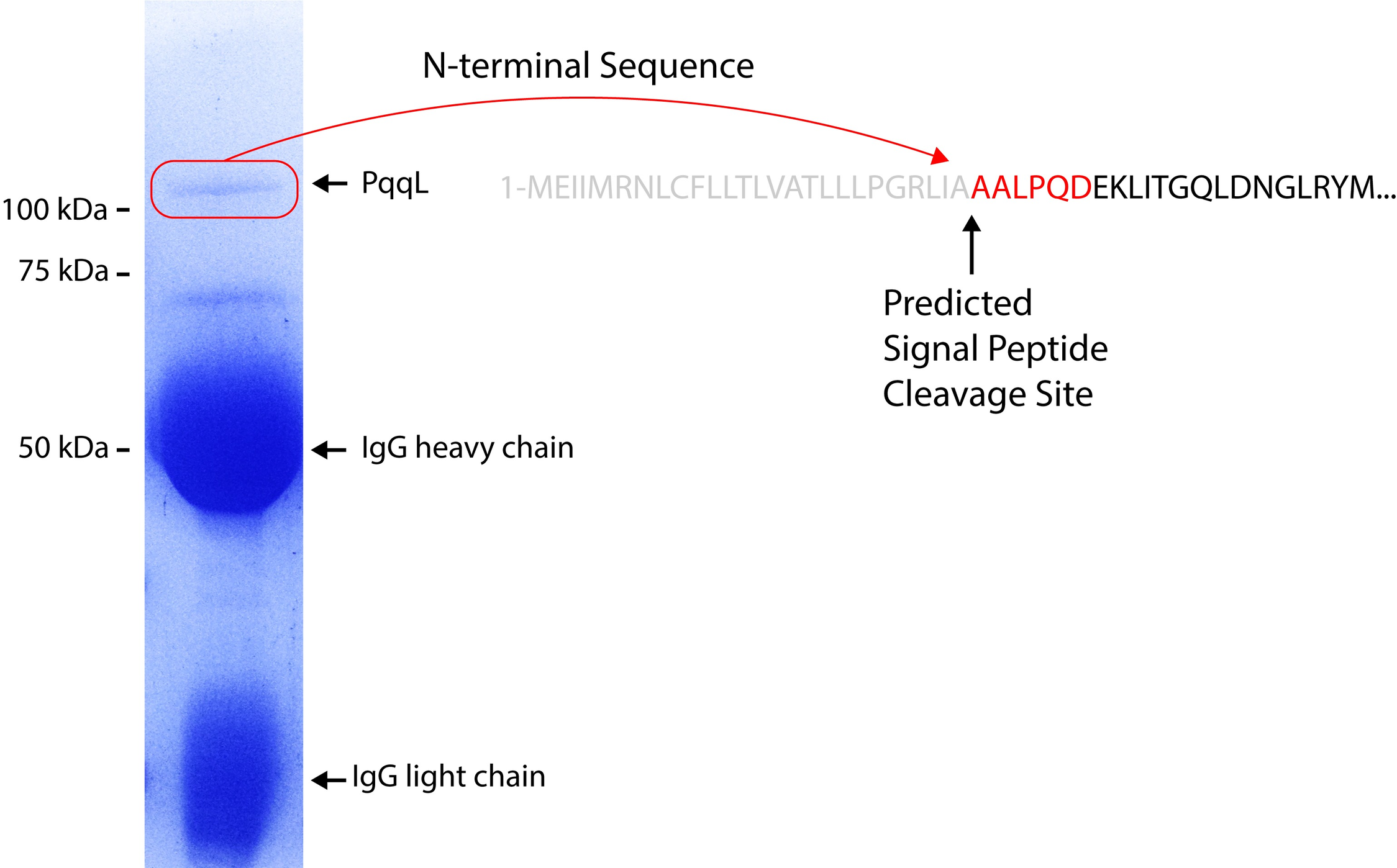

Supplement: S5 Fig — PqqL immunoprecipitated using anti-PqqL serum was isolated via SDS page (left) and N-terminally sequenced using Edman degradation. The sequence of the corresponding band (AALPQD) is consistent with the N-terminal sequence of PqqL after cleavage of its predicted signal peptide. (TIF) [file pgen.1008435.s005.tif]

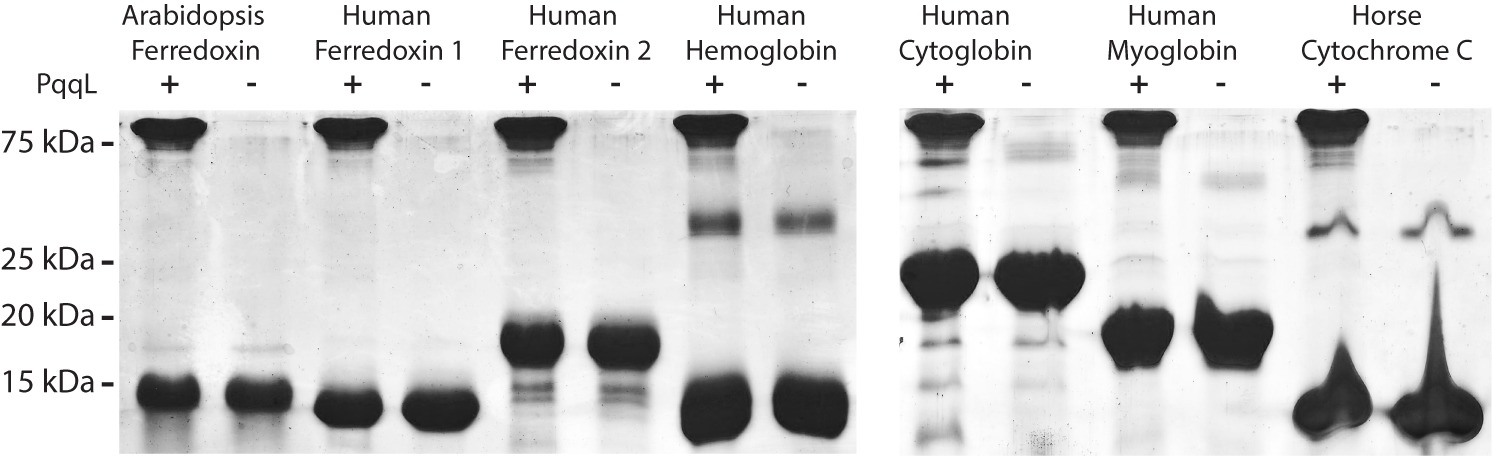

Supplement: S6 Fig — Coomassie brilliant blue stained SDS-PAGE gel visualisation of protease cleavage reactions containing various small iron containing proteins in the presence and absence of PqqL. No proteolytic cleavage by PqqL was observed in these substrates. (TIF) [file pgen.1008435.s006.tif]

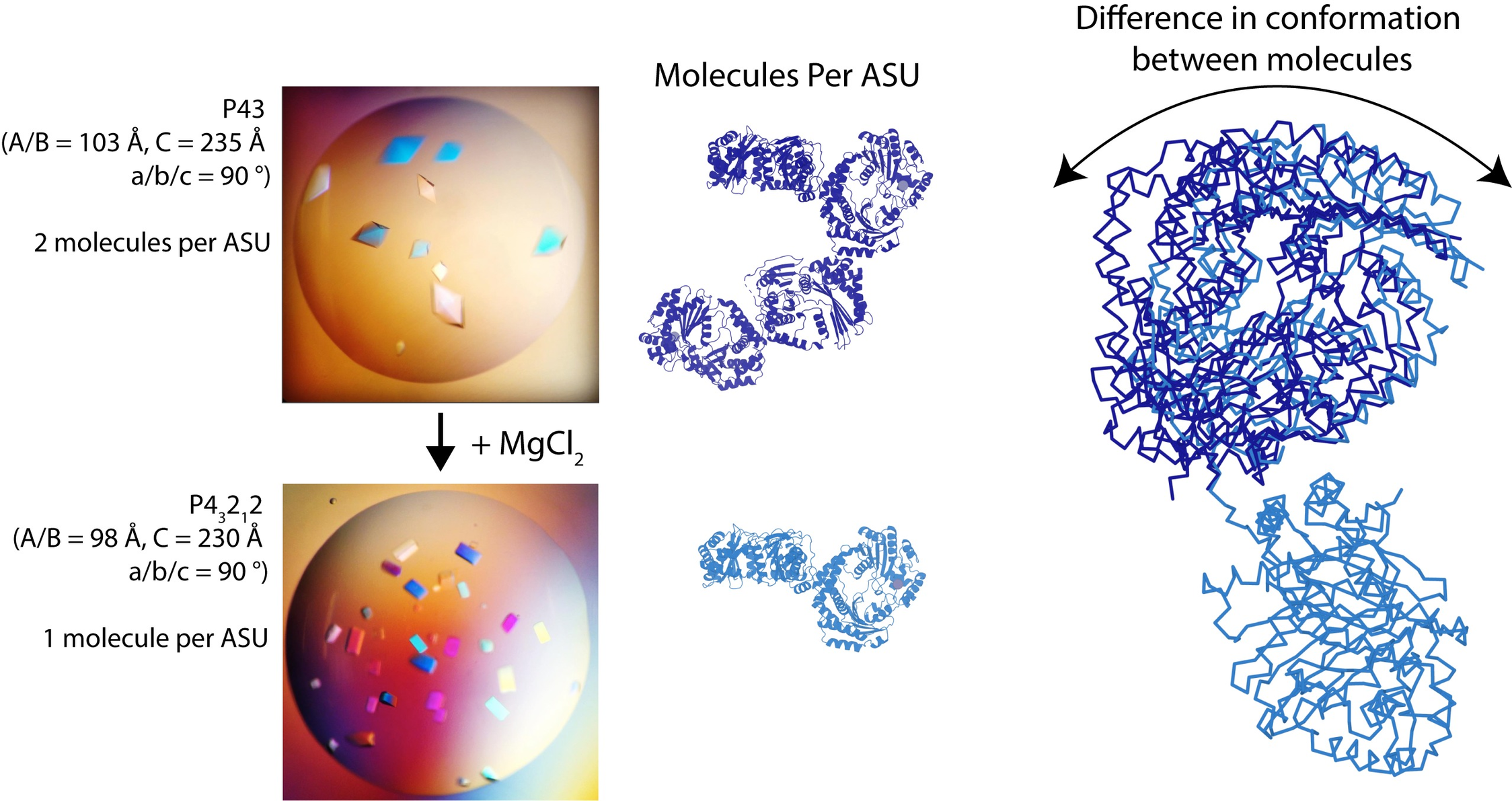

Supplement: S7 Fig — (A) In the absence of MgCl2 PqqL formed poorly diffracting crystals in the space group P43, the addition of MgCl2 led to an increase in symmetry and change in space group to P43212. (B) PqqL molecules in crystals of the space group P43 exhibited a difference in conformation between their two domains, indicative of inherent flexibility of PqqL. (TIF) [file pgen.1008435.s007.tif]
