# Supplementary material for: Protease-associated import systems are widespread in Gram-negative bacteria
Source: PLoS Genet. 2019 Oct 15;15(10):e1008435. doi: 10.1371/journal.pgen.1008435 (PMC6793856; doi:10.1371/journal.pgen.1008435)
Supplement: Attachment — Submitted filename: Reply to reviewers comments.pdf [file pgen.1008435.s015.pdf]

Editor Responses:

Thank you very much for submitting your Research Article entitled 'Protease-associated import systems are widespread in Gram-negative bacteria' to PLOS Genetics. Your manuscript was fully evaluated at the editorial level and by independent peer reviewers. The reviewers appreciated the attention to an important problem, but raised some substantial concerns about the current manuscript. Based on the reviews, we will not be able to accept this version of the manuscript, but we would be willing to review again a much-revised version. We cannot, of course, promise publication at that time.

As you will see from their comments, the three reviewers agreed that this is an interesting study that is clearly written and that it presents new insight into this protease-associated import system. However the three reviewers agreed also that the manuscript is missing a bit more experimental and biological results to support the structural work and its resultant conclusions. Each of the reviewers pointed to missing experiments.

We thank Prof. Buchrieser and Prof. Casadesús for their acknowledgement of the importance and interest of our work and for the constructive suggestion to improve our manuscript, based on the reviewer comments. We feel that we have fully addressed these suggestions and the concerns of the reviewers in the revised version of the manuscript, either through additional experimental work or through reference to relevant studies in the literature. Specifically:

For instance the authors should confirm that that the Ab does not bind to similar region of FusC,

While E. coli does not contain FusC, we have shown that our PqqL antibody is specific, with no band observed for PqqL in a western blot of E. coli ΔpqqL.

They should determine if (separate, non-polar) mutations in YddB and PqqL impair the ability of E. coli to grow in low-iron, as this would be indicative of a role in iron import,

We have shown that the loss of YddB and PqqL does not impair the growth of E. coli in low-iron, when grown under lab conditions.

cellular fractionation, immunoblotting should be done for YddB as it was for PqqL (Fig 4B) and/or microscopy for surface localization.

Due to our inability to raise and antibody to YddB, this experiment was not possible. However, we now reference a number of studies that detect YddB in the outer membrane of E. coli. In addition, we draw attention to the structural features of YddB that indicate outer membrane localisation.

Furthermore the Western blots should be quantified

We have now quantified all western blots presented

and crystallographic statistics after anisotropy correction for YddB, especially cc1/2 and I/s and the validation reports for all the structures, need to be

provided.

We have revised the crystallographic data for the study and now present all relevant/requested statistics.

Comments to the Authors:

Reviewer #1:

Grinder and colleagues are investigating the structure and function of the uncharacterised proteins YddB and PqqL from Ecoli that show some similarity to the FusC and FusA proteins that are involved in iron acquisition from ferredoxin. FusA and FusC encode for an outer membrane receptor that uptakes ferredoxin whereas FusC is a peptidase that releases iron from ferredoxin. Using sequence analysis they show that YddB and PqqL show similarity to the Fus system. Cell growth assays under limiting iron conditions shows that the proteins are upregulated. They also solved the crystal structure of both proteins and they show very similar fold to the Fus proteins suggesting that they are likely distant homologues. Functional data, revealed that YddB has protease activity.

Overall, they provide good evidence that these systems are well conserved among proteobacteria.

We thank the reviewer for the positive assessment of our work and constructive comments regarding the manuscript.

Some issues that need to be addressed:

1. The authors show that under limit iron conditions, the PqqL and YddB are over expressed. Considering the high degree of similarity between PqqL and FusC, can they be confident that the Ab does not bind to similar region of FusC. A control experiment whereas a knockout of PqqL in the presence of Abs should be performed to strengthen this claim. They should also provide information on how the Abs were raised.

Possibly the reviewer misunderstood here. FusC is present in Pectobacterium spp., but not in E. coli. E. coli possesses PqqL/YddB instead of FusC/FusA, not in addition to FusC/FusA. Therefore, the E. coli cell extracts tested in this work to not contain FusC.

Keeping this in mind, we agree with the reviewer that in order to confirm that the PqqL antibody we raised is specifically detecting PqqL, a western blot of extracts from an ΔpqqL strain is a good control. We have now performed this experiment, which confirms that no band corresponding to PqqL is present in the ΔpqqL strain and included it in Figure S4, in the revised manuscript.

We have included the details of PqqL antibody production in the methods under 'Reagents and Antisera generation' of the revised manuscript.

2. The authors have nicely shown that PqqL displays protease activity using a peptide screen. Since they claim that this system is homologous to FusC, why not perform the activity in the presence of ferredoxin? Do the identified peptide sequences much the ferredoxin sequence that they could map them on?

We have now tested the activity of PqqL against a panel of small iron containing proteins, including plant ferredoxin, human ferredoxins I and II and a number of globins. Despite its activity in the peptide assay, PqqL exhibited no activity towards these proteins, suggesting they are not the substrate of YddB/PqqL. These data are shown in Figure S6 of the revised manuscript. This finding is consistent with the high specificity of FusC towards its ferredoxin substrate shown with work published by Mosbahi et al (2018)[1].

*Interestingly, the peptides we identified as being cleaved by FusC in our peptide assay do not correspond to the sequence of the FusC cleavage sites in ferredoxin. It is worth noting that the two sites on ferredoxin cleaved by FusC also do not share the same sequence being, EAG*IDL and QSF*LDD. As discussed in this paper and our previous work, we feel that the cleavage mechanism of this family of proteins is likely to be complex and the sequence specificity of these proteases may vary based on the conformation of both substrate and protease. Clearly further investigation of the mechanisms of cleavage of these proteins will yield exciting results.*

3. I am concerned with the high Rmerge values for all data sets at low resolution. Is it possible that the data suffer from pseudo symmetry? Is the redundancy for PqqL full length really 125?

Despite its long use as a standard for judging the quality of X-ray diffraction data, Rmerge is not a good measure of data quality as it increases relative to data redundancy. To avoid arbitrarily discarding useful high resolution data, it was recommended by experts in data processing that it be abandoned as means of determining where to truncate diffraction data [2, 3]. For data collected in this study we have employed a dual cutoff of CC1/2 >0.5, I/sigma =/>1.5. The higher than expected Rmerge values result from the redundancy of the data and the inclusion of these weak reflections, no pseudo symmetry detected in our data.

The redundancy for PqqL is indeed 125. At the time of collection of these data, we didn't have sufficient phasing power to solve the structure by molecular replacement. We attempted extensive derivatisation with heavy atoms and tried to grow selenomethione labelled crystals but with no success. The high redundancy of the data was intended to maximise the anomalous signal from the Zn we hypothesized to be present in the enzyme active site. Ultimately the phasing power from this Zn atom also proved insufficient, however all the data collected was of high quality so we elected to keep it in

the final dataset. As outlined in the methods, the structure was eventually solved by crystallising a trypsinised fragment of the protein (roughly the first half), solving the structure of that by MR, and using this structure for molecular replacement of the full length protein.

The authors should provide crystallographic statistics after anisotropy correction for YddB, especially cc1/2 and I/s.

In the initial processing of the YddB data we applied anisotropic correction to the data after it had been scaled and merged. This does not allow for reporting of cc1/2 and I/s for the anisotropic correction. In response to the reviewer's comment, we reprocessed the original data in XDS and scaled it using XSCALE. We submitted these unmerged data to the anisotropy server, however we were unhappy with the quality of the maps resulting from the data processed this way. As such, we have elected to process the data to a new resolution cutoff of 2.4 angstrom and not to apply anisotropy correction. As the anisotropy of these crystals isn't extreme, we are discarding a minimum of useful data this way and the maps are of high quality. We have re-refined our structure with these data and amended the PDB deposition. The crystallography data statistics table has been updated accordingly.

In the revision they should provide the validation reports for all the structures. They should also list Ramachandran statistics.

These data have been included with the current submission.

Reviewer #2:

Grinter et al. recently discovered and characterized a unique class of protein import systems dedicated iron uptake from ferredoxin in Pectinobacterium. The system consists of an outer membrane TonB-dependent porin FusA and a periplasmic protease FusC. Here, by using a combination of bioinformatic, biochemical and structural analyses, these authors show the presence of functionally analogous and structurally similar systems in a range of proteobacteria. To support their bioinformatics analysis, they determined the structure of a related system from Escherichia coli comprising the outer membrane component YddB and a periplasmic protease PqqL. They show that PqqL is induced upon iron limitation in E. coli, supporting the role of the system in iron scavenging from an iron-containing protein. PqqL structure determination and its comparison to that of FusC reveals a protein composed of two domains connected by a short linker. These domains adopt a closed conformation in the presence of substrate and an extended one in its absence. This conformational transition is thoroughly characterized by SAXS and molecular dynamics analysis in PqqL and FusC. The authors also determine the substrate specificity of PqqL and demonstrate that it is rather narrow, in line with its role in cleavage of a specific substrate.
The study is original, well executed and the article is very clearly written. The study reveals important information on this new class of systems, by showing that many proteobacteria have the capacity to take up proteins from the environment. The study therefore provides a basis for a vast field of research

that might reveal other biological functions of these protein import systems.

We thank the reviewer for their interest in our work and their positive comments regarding our manuscript. We agree that this study forms the basis for a field of research on protein import in Gram-negative bacteria.

Minor comments:

1. The PqqL western blots appear to be nonlinear and there is a clear difference in protein and control levels between the two strains in Fig. 4A. The recent guidelines require that the linear range of detection be determined and the Western blots be quantified to support the claim that there is more PqqL in urine than in the presence of BiP. The loaded samples correspond to how many bacteria?

The western blotting for the original study was visualised using X-ray film, a number of exposures were collected for each blot. SurA bands from different film exposure lengths were shown for CFT073 and BW25113 in the original figure, leading to the apparent differences in the levels of this control. Quantification from these original blots was difficult due to the overlap of the SurA control band and the lower non-target band. Therefore, we repeated this experiment and visualised the blots using a digital CCD-camera detection. To ensure the expression levels we observed were representative, we performed 3 biological replicates of this experiment. We quantified these blots ensuring that band intensity did not saturate the detector and have presented these data in a new experiment. Over the course of these experiments, while PqqL protein levels in E. coli CFT073 were generally higher than BW25113 when grown in urine, they were variable. Thus we have revised the claim that CFT073 differentially expressed PqqL in urine from the current version of the manuscript. We now only make the general claim that PqqL levels are higher under iron limiting conditions. 4.8×10^7 cells were used for each sample in these blots, after normalisation by absorbance at OD600 nm, serial dilution and colony counting was performed to determine cell numbers.

We have also quantified the intensity of bands from the original localisation blot we performed from scanned X-ray film.

These new data are presented in Figure 4.

2. I suggest that the ion in Fig. 4C be depicted with a different color for better contrast. In addition, the color of the zoomed area in Fig. 4D should be same as in Fig. 4C.

This change has been made to the manuscript, the Zn ion in Figure 4 is now orange.

3. Could the authors describe what was their positive control in the FRET assay for peptide specificity?

There isn't a positive control in this assay as it screens for the presence or absence of peptidase activity. Wells containing pools of peptide that aren't cleaved upon addition of the protease don't emit a FRET signal, while those in which peptides are cleaved do. The activity of the protease against individual peptides from the original pool is then confirmed in an individual FRET assay.

4. Lines 133-139. The authors advance a claim that bacteria containing these protein import systems tend to associate with plant or animal hosts, a claim immediately contradicted by their presence in marine bacteria. It may be better to avoid any general claims at this point as too little is known about their functions or "specific lifestyles". As for most TonB-dependent transporters, these systems are likely to promote uptake of scarce nutrients from the environment.

We agree with the reviewer that stating these systems are generally associated with plant and animal hosts is over interpretation of the available data. We have revised the manuscript (lines 132-139) with the following text to moderate this claim:

'Genome metadata was mined to determine the environment from which the bacteria had been isolated, showing they adopt a variety of lifestyles, which tend to correlate with sequence cluster (Table S1). For example, members of the clusters defined by Pectobacterium and Hemophillus sequences adopt a commensal or pathogenic relationships with plant or animal hosts, while members of the Marinomonas, Marinobacter and Pseudoalteromonas clusters were isolated from marine or other environmental samples (Table S1).'

<u>Other minor text comments:</u>

line 71: … a bacterium …

Corrected

l. 163: …distinct from …

Corrected

l. 476: Cells were…

Corrected

SI legends: l. 727: similarly distant from…

Corrected

l. 734: Structurally distinct…

Corrected

l. 736: remove “the siderophores” at the end of the line.

Corrected

l. 741: … N-terminally sequenced…

Corrected

l. 755: sequence identity of different members…

Corrected

Reviewer #3: Previous work done with Pectobacterium spp. had shown that the outer membrane protein FusA and the periplasmic protein FusC proteins conjoin to import and then degrade ferredoxin as a means toward iron assimilation. In this very interesting follow-up, Grinter et al show that i) gene clusters (proteins) related to FusA/FusC exist in many types of Proteobacteria, ii) the structure of the E. coli protein YddB is similar to that of FusA, and iii) E. coli PqqL is a periplasmic protease that is induced by low-iron growth conditions and is structurally similar to FusC. The MS is very well written and interesting. The structural biology work that was done is especially impressive. The MS’ conclusions are generally appropriate. Thus, the findings here have implications for many Gram-negative’s, including both plant and animal pathogens. However, the MS would benefit from the inclusion of more “biology” (points 1 & 2) and genetic analysis (point 3), in order to strengthen the conclusions made.

We thank the reviewer for their interest in our work and the positive suggestions regarding our manuscript. We have incorporated the suggested changes into the revised version.

Major points

1. Given that the homologs of YddB and PqqL are involved in iron assimilation and that the current study finds PqqL to be more highly expressed in low-iron, there should be some attempt to determine whether YddB-PqqL promotes iron assimilation in E. coli. (That past work by others had shown that YddB is important in systemic infection by a strain of UPEC does not alone make this point.) It is true, as the authors mention in their Discussion, that the substrate for the system need not be the same as that of the Pectobacterium system (i.e., ferredoxin); however, at the least, the authors should determine if (separate, non-polar) mutations in YddB and PqqL impair the ability of E. coli to grow in low-iron, as this would be indicative of a role in iron import. The fact that PqqL is hyper-expressed in LB containing the iron chelator BP (Fig 4A) strongly suggests that the proteins are needed under these growth conditions. It might be necessary to mutate yddB and pqqL in a strain that is lacking

siderophore in order to clearly / dramatically see a role for YddB and PqqL. These experiments are worthwhile even if they do not reveal a link to growth in low-iron, as this would provide evidence for an import that is rather distinct from the Pectobacterium system.

We agree with the reviewer that the specific role that YddB/PqqL plays in iron acquisition is a fascinating question. We have performed the following experiments based on the reviewer's suggestions and included the data in Figure 5 of the current form of the manuscript:

- *We have constructed separate non-polar deletions of PqqL and YddB and shown they do not affect the growth of E. coli in LB media +/- 2,2'bipyridine.*
- *We have tested the effect of deletion of YddB on growth of a strain lacking all TBDTs associated with iron acquisition and shown that it does not cause a further growth defect in LB media +/- 2'2,bipyridine.*

These data and included in Figure 5 of the revised manuscript and show no growth defect associated with YddB/PqqL under these conditions, suggesting this system does not play a general role in iron acquisition in E. coli. We feel that it is likely the substrate protein for this system is present in a specific host or environmental niche occupied by E. coli and while it is upregulated generally in response to iron limitation, it is unable to support growth under lab conditions as the substrate protein is not present.

2. Lines 147-156. Although it was shown later in the MS that PqqL is localized to the periplasm compatible with its role as an analog of FusC, the outer membrane / surface localization of YddB was not documented. But, it should have been, given the (implied) conclusion that YddB is an outer membrane transporter analogous to FusA. Cellular fractionation and immunoblotting could be done as it was for PqqL (Fig 4B) and/or microscopy for surface localization.

We agree with the reviewer determining the localisation of YddB directly by immunoblotting or immunofluorescence microscopy would be a nice experiment. To this end we twice attempted to raise an antibody to YddB in rabbits. One attempt was utilising purified YddB inclusion bodies and the other with purified natively folded protein. Neither attempted produced antisera with reactivity to YddB. Based on practical and ethical considerations we elected at this point not to pursue the generation of an antibody for this protein further.

Despite the lack of this data there is strong theoretical and experimental evidence for the localisation of YddB to the outer membrane:

Firstly, YddB is definitively a membrane protein. We purified it from solubilised membranes in the presence of detergent and its crystal structure clearly shows the presence of a transmembrane region with visible electron density for shielding detergent molecules. This information is now included in the manuscript in Figure S2 and lines 164-165 of the revised manuscript:

'As with FusA, YddB possesses a 22-stranded β-barrel fold, the pore of which is occluded by a globular N-terminal plug-domain. This fold is characteristic of the integral outer membrane TBDT family and like these proteins YddB possesses a hydrophobic transmembrane region (Figure S2).'

Secondly, YddB belongs to the TonB-dependent transporter (TBDT) family, which is a well characterised superfamily of outer membrane proteins [4]. YddB's closest characterised homologue the TBDT FusA has been shown to act as a conduit of ferredoxin and related bacteriocins (pectocins) across the outer membrane [1, 5]. The β-barrel architecture of TBDTs, shared by YddB, is characteristic of outer-membrane proteins, with inner membrane proteins being characteristically alpha-helical [6]. As implied by the name the of TBDT family, these transporters depend of energy provided by the TonB-ExbBD complex. This complex resides in the inner membrane and the TonB-component spans the periplasm to interact with the TBDT in the outer membrane. This interaction provides energy from the proton motive force to drive substrate import through the TBDT and across the outer membrane[7]. Thus it is highly unlikely that a protein with this architecture would be located anywhere other than the outer membrane.

Thirdly, experimental data from a number of studies confirms the outer membrane localisation of YddB. Utilising a proteomics approach, a studies by Martorana et al. in 2014 [8] and Vertommen et. al. 2009 [9], showed YddB to be present in outer membrane fraction from E. coli cells. Moreover, three studies characterising the membrane protein content of E. coli outer membrane vesicles identified YddB as present in OMVs [10-12]. These studies provide strong experimental evidence for the outer membrane localisation of YddB.

We have included the following text on lines 149-151:

'YddB belongs to the outer membrane localised TBDT family and has been detected in the outer membrane and in outer membrane vesicles of E. coli in a number of studies [16-20].'

And added references to these articles to provide the reader with a clear picture of the evidence supporting YddB's outer membrane localisation.

3. Lines 171, 182-184. Given that levels of PqqL are increased in low-iron growth conditions, it should be determined and discussed whether the yddB/pqqL operon is iron-regulated and Fur-regulated. Basic qRT-PCR can determine if the genes are iron-regulated, and sequence analysis should be able to identify a putative Fur box. (The fact that the operon was shown by others to be upregulated in urine does not alone make this point.). Following on point 2, immunoblotting could then confirm whether YddB levels are also influenced by iron levels.

An excellent study by Seo et. al. in 2014 [13], comprehensively determined the Fur regulatory network in E. coli. This study found that the yddA/yddB operon is repressed by Fur under iron replete conditions, with the Fur shown

by CHIP-seq to bind at a number of sites in the promoter region of the operon. The yddA/yddB operon was shown to include pqqL by Subashchandrabose et. al. in 2013 [14], confirming that both yddB and pqqL are directly regulated by Fur at the transcriptional level. Fur regulation of the ydd/pqqL operon was also reported by an earlier study, and a Fur box upstream of yddA was identified [15].

It is our opinion that these published data make the suggested qRT-PCR experiments unnecessary. However, we agree with the reviewer that it is important to clearly outline the Fur mediated iron dependent regulation of yddA/yddB/pqqL in the manuscript. As such we have revised the manuscript with the following text at lines 151-154:

'YddB is encoded in an operon with the FusC homologue PqqL, which has been shown to be expressed in response to iron limitation, to be regulated by the ferric uptake regulator (Fur), and to be important in systemic infection of uropathogenic E. coli in a mouse model of infection [21-23].'

Lines 183-184:

'The first of these hypotheses is supported by the fact that the ydd/pqqL operon has been shown to be regulated by Fur and is upregulated under iron limiting conditions [22, 23].'

and lines 242-243:

'The transcriptional upregulation of the ydd/pqql operon and increased expression of PqqL under iron limitation suggest that this operon may play a general role in iron acquisition [22].'

to highlight these data.

Minor points

1. Lines 220-221, 260. Materials & Methods needs a section on how FusC was obtained and used.

The following 'FusC for peptide cleavage assays and structural analysis was purified as previously described [6].' Has been added to line 438-439 of the revised manuscript.

2. Line 338. YddB is missing from the section header.

Corrected. Section now reads 'Protein Expression and Purification'

3. Lines 341 464. Insert references to Table S6.

Included in the revised manuscript

4. Lines 354, 370, 376. Make clearer how the fractions of interest were

identified.

Fractions were identified based on absorbance at 280 nm. As both proteins expressed reasonably well, the peak corresponding to recombinant protein was obvious based on magnitude. This has been clarified in the current version of the manuscript

5. Line 426. Provide a reference and source for this reagent and method.

Included in the revised manuscript

6. Lines 466-467. Provide a source for the human urine used.

Included in the revised manuscript (Line 570)

7. Lines 491-492. Provide the source and if needed methods used to obtain these five antisera.

Included in the revised manuscript in methods under 'Reagents and Antisera generation'

8. Lines 522-651. The formatting of the references is not consistent.

Corrected in the current manuscript

9. Lines 689, 692. Should this read "BW251113" rather than "K12"?

Corrected in the current manuscript

10. Lines 138, 727, 735, 755. Check for typos and word usage.

Corrected in the current manuscript

References:

1. Mosbahi K, Wojnowska M, Albalat A, Walker D. Bacterial iron acquisition mediated by outer membrane translocation and cleavage of a host protein. Proceedings of the National Academy of Sciences. 2018:201800672.
2. Evans PR, Murshudov GN. How good are my data and what is the resolution? Acta Crystallographica Section D: Biological Crystallography. 2013;69(7):1204-14.

3. Karplus PA, Diederichs K. Linking crystallographic model and data quality. Science. 2012;336(6084):1030-3.
4. Noinaj N, Guillier M, Barnard, Travis J., Buchanan SK. TonB-Dependent Transporters: Regulation, Structure, and Function. Annu Rev Microbiol. 2010;64(1):43-60. doi: doi:10.1146/annurev.micro.112408.134247.
5. Grinter R, Josts I, Mosbahi K, Roszak AW, Cogdell RJ, Bonvin AM, et al. Structure of the bacterial plant-ferredoxin receptor FusA. Nat Commun. 2016;7.
6. Wimley WC. The versatile β-barrel membrane protein. Current opinion in structural biology. 2003;13(4):404-11.
7. Hickman SJ, Cooper RE, Bellucci L, Paci E, Brockwell DJ. Gating of TonB-dependent transporters by substrate-specific forced remodelling. Nat Commun. 2017;8.
8. Martorana AM, Motta S, Di Silvestre D, Falchi F, Dehò G, Mauri P, et al. Dissecting *Escherichia coli* outer membrane biogenesis using differential proteomics. PLoS ONE. 2014;9(6):e100941.
9. Vertommen D, Ruiz N, Leverrier P, Silhavy TJ, Collet JF. Characterization of the role of the *Escherichia coli* periplasmic chaperone SurA using differential proteomics. Proteomics. 2009;9(9):2432-43.
10. Lee EY, Bang JY, Park GW, Choi DS, Kang JS, Kim HJ, et al. Global proteomic profiling of native outer membrane vesicles derived from *Escherichia coli.* Proteomics. 2007;7(17):3143-53.
11. Scorza FB, Doro F, Rodríguez-Ortega MJ, Stella M, Liberatori S, Taddei AR, et al. Proteomics characterization of outer membrane vesicles from the extraintestinal pathogenic *Escherichia coli* ΔtolR IHE3034 mutant. Mol Cell Proteomics. 2008;7(3):473-85.
12. Wurpel DJ, Moriel DG, Totsika M, Easton DM, Schembri MA. Comparative analysis of the uropathogenic *Escherichia coli* surface proteome by tandem mass-spectrometry of artificially induced outer membrane vesicles. Journal of proteomics. 2015;115:93-106.
13. Seo SW, Kim D, Latif H, O'Brien EJ, Szubin R, Palsson BO. Deciphering Fur transcriptional regulatory network highlights its complex role beyond iron metabolism in *Escherichia coli*. Nat Commun. 2014;5:4910.
14. Subashchandrabose S, Smith SN, Spurbeck RR, Kole MM, Mobley HLT. Genome-Wide Detection of Fitness Genes in Uropathogenic *Escherichia coli* during Systemic Infection. PLoS Pathog. 2013;9(12):e1003788. doi: 10.1371/journal.ppat.1003788.
15. McHugh JP, Rodríguez-Quiñones F, Abdul-Tehrani H, Svistunenko DA, Poole RK, Cooper CE, et al. Global iron-dependent gene regulation in *Escherichia coli* A new mechanism for iron homeostasis. J Biol Chem. 2003;278(32):29478-86.
